# A functional *PTPN22 *polymorphism associated with several autoimmune diseases is not associated with IgA deficiency in the Spanish population

**DOI:** 10.1186/1471-2350-7-25

**Published:** 2006-03-15

**Authors:** Concepción Núñez, Raquel López-Mejías, Alfonso Martínez, M Cruz García-Rodríguez, Miguel Fernández-Arquero, Emilio G de la Concha, Elena Urcelay

**Affiliations:** 1 Department of Clinical Immunology, Hospital Clínico San Carlos, Madrid, Spain; 2 Department of Immunology, Hospital Universitario La Paz, Madrid, Spain

## Abstract

**Background:**

The 1858C/T SNP of the *PTPN22 *gene has been associated with many autoimmune diseases, suggesting the existence of an inflammatory process common to all of them. We studied the association of that polymorphism with immunoglobulin A deficiency (IgAD) following a double approach: a case-control and a TDT study.

**Methods:**

A total of 259 IgAD patients and 455 unrelated matched controls, and 128 families were used for each approach. Comparisons were performed using Chi-Square tests or Fisher's exact test when necessary.

**Results:**

No association between the *PTPN22 *1858C/T SNP and IgA deficiency was found in any case (allelic frequencies 8% vs. 6% in patients and controls, respectively, OR= 1.14 (0.72–1.79), p= 0.56; TDT p = 0.08).

**Conclusion:**

The result obtained seems to reinforce the consideration of IgA deficiency as a primary immunodeficiency rather than an autoimmune disease.

## Background

Selective IgA deficiency (IgAD) is the most prevalent primary immunodeficiency in white populations, with values around one out of 600 [1]. This disease is characterized by a severe deficiency or total absence of IgA class immunoglobulins in the serum and secretions. Their clinical symptoms are very variable, and thus some IgAD patients are relatively healthy while others show significant illness, mainly a higher susceptibility to infections, autoimmune diseases and allergies. IgAD shows a multifactorial origin, with genetic and environmental factors involved. Class II and class III HLA genes have been implicated in the origin of this disease (see [2] and references therein). However, these HLA genetic components can not explain the totality of the genetic influence in this disease.

The molecular bases underlying the origin of IgAD are not completely understood. Although it has been recently shown that a mutation in TACI is involved in the origin of the disease in some patients [3], other genetic and environmental factors remain to be discovered. Despite IgAD has been traditionally considered as an immunodeficiency, some authors [4] have pointed out that it could be an autoimmune disease.

Recently, a single-nucleotide polymorphism (SNP) of the *PTPN22 *(protein tyrosine phosphatase, non-receptor type 22) gene, 1858C/T, has been found associated with many autoimmune diseases [5,6]. This gene, located on chromosome 1p13, encodes a lymphoid protein tyrosine phosphatase (PTP Lyp) involved in the regulation of the T-cell receptor signaling pathway. The 1858C/T SNP results in an amino-acid change that alters the function of the PTP Lyp. The allele 1858T originates an altered protein unable to inhibit T-cell responses, what would imply a pathogenic response.

The aim of this work is to study the association of the 1858C/T polymorphism of the *PTPN22 *gene with IgAD and to deepen in its implication concerning the controversial origin of this disease.

## Methods

### Subjects

We studied a total of 259 white patients with IgAD as defined by the World Health Organization Group on Primary Immunodeficiencies (<0.05 g/L [7]), recruited from a single Spanish Centre (Hospital La Paz, Madrid) and 455 unrelated matched healthy controls obtained from blood donors at the Hospital Clínico San Carlos (Madrid). One hundred and twenty-eight families composed of the affected subject and their parents were also included in the study. Informed consent was obtained from all the participants. This study was approved by the Ethics Committee of the Hospital Clínico San Carlos.

### Genotyping

All the individuals included in the study were typed for the *PTPN22 *1858C/T (rs2476601) variant using a TaqMan 5' allelic discrimination Assay-By-Design method (Applied Biosystems, Norwalk, USA).

HLA typing was performed as previously described [2].

### Statistical analysis

Allelic frequencies and carriage rates were compared between cases and controls by means of chi-square tests or Fisher's exact test when necessary (expected values below 5). Statistical analyses were performed with the statistical package EpiInfo v5.00 (CDC, Atlanta, USA). Considering our sample size and allelic frequencies, the statistical power of our study to detect the influence of 1858C/T is 80% for an OR = 1.8 [8].

Family data were analyzed using the Transmission Disequilibrium Test (TDT) [9]. Obviously, only information from heterozygous parents was considered because only in them we can differentiate between the transmitted and non-transmitted allele.

## Results

Allelic and genotypic frequencies for the case-control study are shown in Table 1. Allelic frequencies in controls are significantly different to those previously described in other white populations (p = 0.0012, vs. North American population and p = 0.0015 vs. Sardinian population [10,11]), but previously reported frequencies also differ significantly between them (p = 2.0*10^-7 ^between North American and Sardinian populations). *PTPN22 *1858C/T allelic frequencies do not significantly differ between controls and IgAD patients (p = 0.56). Statistically significant differences were neither observed when carriage rate of T allele was compared between patients and controls (p = 0.56).

**Table 1:** Genotype and allelic PTPN22 frequencies in patients (n = 259) and controls (n = 455).

	IgAD patients (%)	Controls (%)
Genotype		
CC	225 (86.9)	402 (89.4)
CT	32 (12.3)	50 (11.0)
TT	2 (0.8)	3 (0.6)
Allele		
C	482 (93.0)	854 (93.9)
T	36 (7.0)	56 (6.1)

Due to the known association of some HLA alleles with IgAD, we also studied the association between *PTPN22 *and IgAD stratifying by carriage of DRB1*0102, DR3, DR3 and TNFa2b3, and for DR7, separately or combined, in order to look for epistatic interactions between HLA-risk factors and *PTPN22*. No statistically significant results were obtained in any case. The family study also showed negative results. Only 31 families included in this study showed at least one heterozygous progenitor and could, therefore, be used in the TDT. No significant preferential transmission was observed for any of the alleles of this gene, although a small trend was observed for the allele 1858C (allele C: 22 transmitted vs. 11 non-transmitted; p = 0.08). Similar results were obtained when the data corresponding to the transmission from the mother and the father were separately considered (data not shown).

## Discussion

*PTPN22 *has been found associated with most autoimmune diseases studied so far, specifically with some of the most prevalent ones, Graves disease, rheumatoid arthritis, systemic lupus erythematosus and type 1 diabetes [6,10-12]. This is the first study analyzing the involvement of the *PTPN22 *gene in IgA deficiency. However, we could not substantiate an association between *PTPN22 *and this disease.

The association of several autoimmune diseases with the *PTPN22 *1858C/T SNP has been considered as indicative of the existence of an inflammatory process common to many autoimmune diseases. According to this idea, the lack of a positive association with that gene would support the non-autoimmune etiology of IgAD. Any defect in some of the complex involved in T/B collaboration might affect immunoglobulin production and therefore be involved in the origin of IgAD. The consideration of IgAD as an immunodeficiency, due to alterations in some process involved in the production of IgA, is generally accepted. However, it has been known for a long time that immunodeficiencies and autoimmune processes occur very often in the same individual [13,14]. As a matter of fact, IgAD patients have autoimmune as well as immunodeficient features. However, perhaps one of those components is developed secondarily to the other; thus the main difficulty is to clarify which one comes first.

In view of our results we can not support firmly any hypothesis, but it seems clear that at least IgAD is not a conventional autoimmune disease, as shown by the lack of association with *PTPN22 *1858T. This indirectly reinforces the original idea about the immunodeficient basis of this disease. Of course, the lack of association with *PTPN22 *is not as conclusive as a positive result, which without doubt would have supported the autoimmune nature of IgAD. Thus, one of the most important challenges for the future research on IgAD will be to clarify this controversy.

## Conclusion

No association between the 1858C/T SNP of the PTPN22 gene and IgA immunodeficiency has been found in a Spanish population. This fact supports the primary immunodeficient etiology of IgAD as opposed to an autoimmune basis.

## Abbreviations

IgA: immunoglobulin A.

IgAD: immunoglobulin A deficiency.

SNP: single-nucleotide polymorphism.

PTP Lyp: lymphoid protein tyrosine phosphatase.

PTPN22: protein tyrosine phosphatase non-receptor type 22.

TDT: transmission disequilibrium test.

HLA: human leukocyte antigen.

MHC: major histocompatibility complex.

TNF: tumor necrosis factor.

## Competing interests

The author(s) declare that they have no competing interests.

## Authors' contributions

CN supervised the genetic analysis, performed the statistical analysis and drafted the manuscript. RLM carried out the genetic analysis of the samples and participated in the statistical analysis. AM participated in the statistical analyses, drafted the manuscript and revised it critically. MCGR participated in IgA quantification and the collection of samples. MFA participated in the collection of samples and participated in the design and coordination of the study. EGC participated in the design of the study and revised critically the manuscript. EU conceived the study, participated in its design and coordination and revised critically the manuscript.

## Pre-publication history

The pre-publication history for this paper can be accessed here:


